# A scale for measuring nursing digital application skills: a development and psychometric testing study

**DOI:** 10.1186/s12912-024-02030-8

**Published:** 2024-05-31

**Authors:** Shijia Qin, Jianzhong Zhang, Xiaomin Sun, Ge Meng, Xinqi Zhuang, Yitong Jia, Wen-Xin Shi, Yin-Ping Zhang

**Affiliations:** 1 Faculty of Nursing, Xi’an Jiaotong University Health Science Center, No.76, West Yanta Road, Xi’an, Shaanxi 710061 China; 2 Department of Nursing, Xi’an No.3 Hospital, The Affiliated Hospital of Northwest University, Xi’an, Shaanxi 710018 China

**Keywords:** Digital skills, Nursing digital application skill, Scale development, Psychometric test

## Abstract

**Background:**

The adoption of digitization has emerged as a new trend in the advancement of healthcare systems. To ensure high-quality care, nurses should possess sufficient skills to assist in the digital transformation of healthcare practices. Suitable tools have seldom been developed to assess nurses’ skills in digital applications. This study aimed to develop the Nursing Digital Application Skill Scale (NDASS) and test its psychometric properties.

**Methods:**

The Nursing Digital Application Skill Scale was developed in three phases. In Phase 1, an item pool was developed based on previous literature and the actual situation of nursing work. Phase 2 included 14 experts’ assessment of content validity and a focus group interview with 30 nurses to pretest the scale. In phase 3, 429 registered nurses were selected from March to June 2023, and item analysis, exploratory factor analysis, and confirmatory factor analysis were used to refine the number of items and explore the factor structure of the scale. Additionally, reliability was determined by internal consistency and test-retest reliability.

**Results:**

The final version of the NDASS consisted of 12 items. The content validity index of NDASS reached 0.975 at an acceptable level. The convergent validity test showed that the average variance extracted value was 0.694 (> 0.5) and the composite reliability value was 0.964 (> 0.7), both of which met the requirements. The principal component analysis resulted in a single-factor structure explaining 74.794% of the total variance. All the fitting indices satisfied the standard based upon confirmatory factor analyses, indicating that the single-factor structure contributed to an ideal model fit. The internal consistency appeared high for the NDASS, reaching a Cronbach’s alpha value of 0.968. The test-retest reliability was 0.740, and the split-half coefficient was 0.935.

**Conclusion:**

The final version of the NDASS, which possesses adequate psychometric properties, is a reliable and effective instrument for nurses to self-assess digital skills in nursing work and for nursing managers in designing nursing digital skill training.

## Background

With the rapid development of digital technologies, we have ushered in the era of digitalization. As a global phenomenon, digitization involves the integration of digital technology into increasingly diverse aspects of professional and personal lives [1]. In healthcare, numerous digital innovations, such as open access to health and treatment information, biomedical research on the Internet, the provision of mobile health services, wearable devices, health information technology, telehealth and telemedicine, have been implemented [2]. These innovations are envisioned to make healthcare more accessible and flexible for the general public, eliminating inequality and inefficiencies in the healthcare system while also enhancing the quality and satisfaction of patient care [2–6]. More importantly, health interventions delivered through digital technologies have proven beneficial in clinical practice, especially in the fields of disease rehabilitation, vaccination, and improvement of psychological problems [7–9]. According to the 14th Five-Year Plan for National Health Informatization issued by the National Health Commission of the People’s Republic of China, digital health services will become an important part of the medical and health service system. It can be seen that digitization has become a new trend in the future development of health systems.

As the cornerstone of the healthcare system, nurses play an indispensable role in providing professional, high-quality, and safe patient care [10]. Previous investigations of the inclusion of digitalization in patient care have focused on the effects and challenges of digital technology applications [11, 12], the economic benefits of digital technologies [13], and the opinions and insights of nurses on digital technology applications [14]. However, another issue that has not been given enough focus is the importance of nurses having sufficient skills to integrate new technologies into clinical practice and use digital technologies effectively in daily work. Nurses with inadequate skills will not be able to use digital technologies appropriately, resulting in an increased incidence of errors and the creation of new safety risks [15]. Negative experiences of technology usage can also influence nurses’ attitudes toward other new technologies [16]. In addition, lower skill levels were reportedly associated with greater technostress, which has a nonnegligible impact on long-term consequences, such as burnout symptoms, job satisfaction, and headaches [17]. Moreover, the COVID-19 pandemic has highlighted the importance of telehealth, which places greater demands on nurses’ digital skills.

Currently, there is no clear definition of digitalization skills possessed by nurses. In its publication *Key Competences for Lifelong Learning*, the European Council defined digital competence as the confident, critical and responsible use of, and engagement with, digital technologies for learning, at work, and for participation in society. An individual who is considered competent in a particular field should possess knowledge, skills, and attitude simultaneously [18]. This study aims to assess the proficiency of nurses in utilizing digital technologies effectively within clinical settings, particularly focusing on their skill mastery in this domain. Therefore, we are more inclined to use “digital skill” instead of “digital competence” to define it. The digital literacy framework for teachers developed by the Ministry of Education of China suggests that digital application refers to the skill of teachers to apply digital technology resources to carry out educational and teaching activities. Referring to the definitions of the Council and the Ministry of Education, this study further defines these skills as nurses’ skills in utilizing digital technologies to carry out nursing work and names these skills “nursing digital application skills”.

Researchers have developed numerous digital skill assessment tools for different populations, including college students, schoolchildren, working professionals, and others [19–22]. These evaluation standards focused on the characteristics of the respective populations and failed to address the professional content of health care. A few instruments for measuring healthcare professionals’ core competencies have incorporated individual items related to the use of digital technologies; however, these instruments cannot provide a comprehensive assessment of digital skills [23, 24]. One study used five items to measure and broadly generalize the digital abilities of health professionals in psychiatric hospitals, but the questions were vague [17]. Other studies have evaluated skills using certain digital technologies, such as electronic health records (EHR) documentation and robots [25, 26]. In addition, the digital health scale developed by Jarva et al. intersects with the elements of nurses’ digital application skills [27]. In this context, this study aimed to develop a reliable and brief scale, namely, the Nursing Digital Application Skill Scale (NDASS), to rapidly evaluate the digital skills required in nursing work.

## Methods

### Research design and methods

**Fig. 1:**
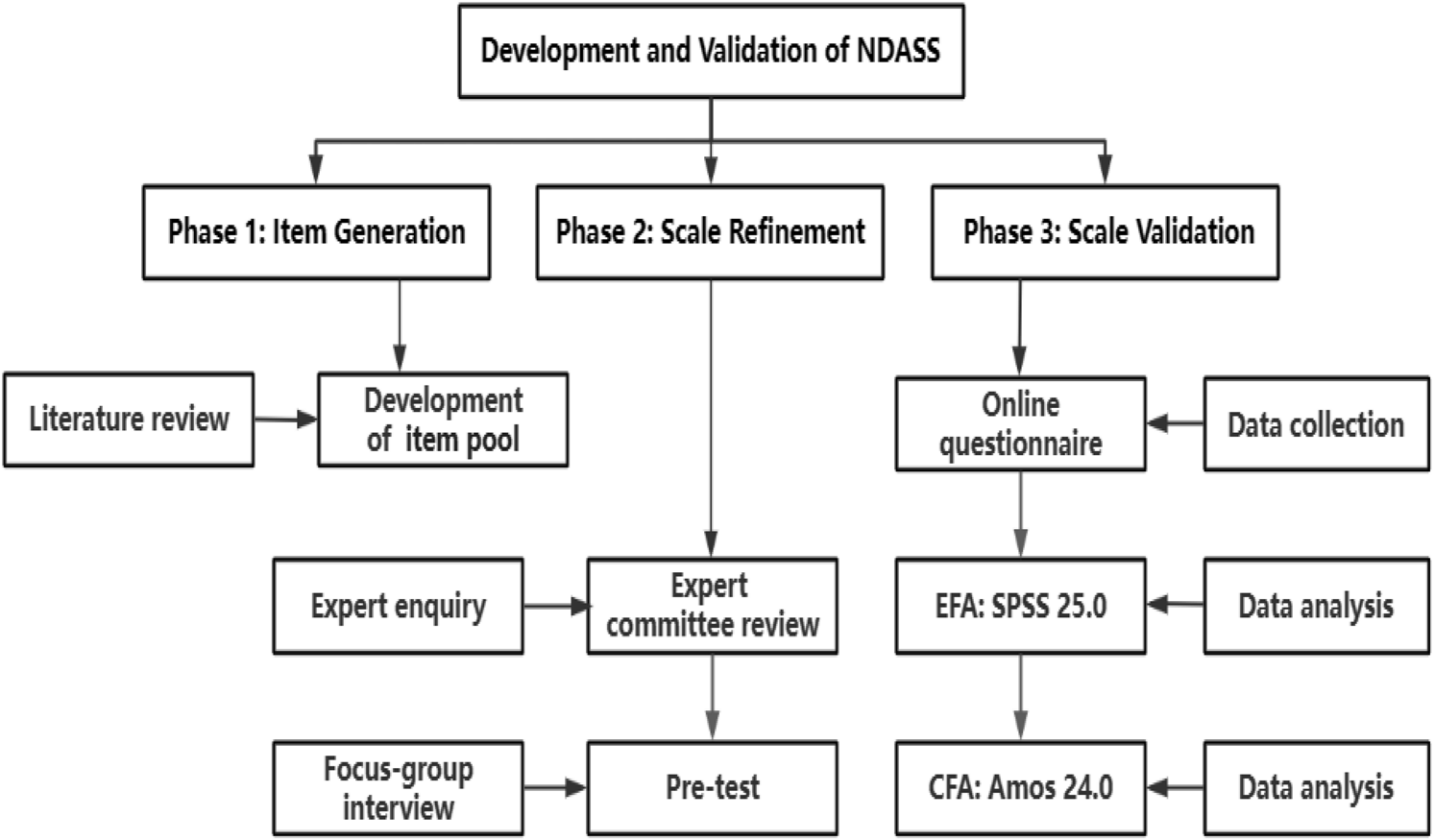
Phases and methods of development and validation of the NDASS

A multistep approach, which included item generation, scale refinement and scale validation, was utilized in this methodological research. In phase 1, the initial item pool was developed based on an extensive literature review. In phase 2, an expert committee review was conducted to evaluate the importance of the items. A pretest was then conducted among a small sample of 30 nurses. The participants completed the scale and provided feedback on the scale’s applicability and acceptance level through a focus group interview. In phase 3, an online questionnaire was used to gather information from participants at several hospitals in Northwest China. After the data were collected, exploratory factor analysis (EFA) was conducted with SPSS 25.0 to determine the internal factor structure of the scale, and confirmatory factor analysis (CFA) was performed with AMOS 24.0 to verify the model-data fit and convergent validity. Figure 1 displays the three phases and different methods used in each phase.

### Item generation

The initial item pool was developed by a research team of five researchers, including a nursing professor, two nursing doctoral students and two nursing students with master’s degree. Through an extensive literature review, the team integrated items related to the application of digital technologies from previous instruments and then removed or merged items with similar meanings. The key terms were (skill OR literacy OR competenc*) AND (“digital technology” OR “digital health” OR “Information Computer Technology” OR informatics OR computer OR internet OR media) AND (nurs* OR “health professional” OR “health care”) AND (scale OR questionnaire OR measure* OR assess* OR evaluat*). The databases Web of Science, PubMed, Medline, China National Knowledge Infrastructure (CNKI) and Wan Fang Data were searched from their inception through September 2022. Only articles in Chinese and English were included.

To accommodate nursing work scenarios, items were rewritten according to the criteria for “nurses’ skills in utilizing digital technologies to carry out nursing work.” On this basis, the expression of the items was unified, and the order of the items was changed to facilitate the reading of participants. The first version of the NDASS was developed with the reference of the Digital Competence Framework for Citizens 2.2 published by the European Commission, based on the questionnaires of Fan et al. [21], van Laar et al. [22], Peart et al. [28], a digital literacy framework for teachers, and so on. As the scale was specifically developed for nurses, we added some items that assess specific digital skills required in the context of clinical nursing according to the evaluation instruments for nurses’ related skills. For example, Item 6, “I can use statistical software to analyze nursing data”, was derived from the Information Literacy Self-rating Scale for Clinical Nurses [29]. The scale was designed with a 5-point Likert scale, with 5 response options available for the items, ranging from 1 for strongly disagree to 5 for strongly agree. The 14 items of the first version of the NDASS are shown in Table 1.

**Table 1:** Measuring items of the initial version of NDASS (14 items)

Items
1. I can integrate and reproduce existing digital content
2. I can create new digital content that meets expectations
3. I can protect intellectual property when creating digital content
4. I can use digital equipment proficiently in nursing work
5. I can use digital technology to analyze nursing problems objectively
6. I can use statistical software to analyze nursing data
7. I can express my thoughts clearly on the Internet
8. I can use digital technology to support nursing decision-making
9. I can use digital technology to promote nurse-patient relationships
10. I can use digital technology to collaborate with others
11. I can use digital technology to participate in social activities
12. I can tolerate different perspectives on the internet
13. I can use digital technology resources for continuous learning
14. I can apply digital technology to promote innovative nursing practices

### Scale refinement

At this stage, the scale was refined through an expert committee review and a pilot study. The preliminary version of the NDASS was reviewed by a panel of 14 independent experts who specialized in nursing management, nursing education, clinical nursing and scale development research. All the experts held deputy senior titles or higher and had an average of more than 25 years of work experience. The experts were invited to judge the importance of each item for content validation using a 5-point scale ranging from 1 (not important) to 5 (very important). Items rated as 4 or 5 suggest that experts have reached a consensus regarding importance. In addition, the experts were also asked to provide specific suggestions for improvements to the scale and each item.

Before applying the reviewed scale to a large sample, we ran a pilot study among a small sample of 30 nurses in Northwest China. These volunteers were recruited through convenience sampling and were asked to complete the scales. The pilot study allowed us to detect problems with wording, terminology, instructions and the clarity of options. A focus group interview was also conducted to explore participants’ perceptions and understanding of the scale and items and to take their advice for improvement. The outline of the interview was as follows: Q1: Do you have any suggestions for the instructions of the scale? Q2: Which items do you find difficult to comprehend? Q3: Do you have any recommendations for the wording of the items? After this process, the official version was developed.

### Participant and setting

A cross-sectional validation study was conducted with 429 registered nurses from various hospitals in Northwest China using convenience sampling. The inclusion criteria for participants were as follows: (a) currently employed in a medical unit; and (b) willing to participate in the study. Participants who took extended leave were excluded. Anonymity and confidentiality were assured, and participants were told that they could withdraw at any point without consequences. From March to June 2023, the data were collected via an online survey utilizing sojump (an online research survey tool; http://www.sojump.com). Approval was obtained from the Ethics Committees of the First Affiliated Hospital of Xi’an Jiaotong University. All procedures followed were in accordance with the ethical standards of the Declaration of Helsinki. Five nurses refused to complete the survey. Thus, we obtained 424 cases for analysis. The 424 cases were randomly split into two groups using SPSS 25.0: one group of 200 cases for EFA and another group of 224 cases for CFA.

### Data analysis

SPSS 25.0 and AMOS 24.0 were used for the data analysis. The statistical description of the demographic variables was carried out by frequency tables, means, and standard deviations (SDs). The content validity index (CVI) was computed to quantify scores for each item and the whole scale. Items rated as 3 (moderately important), 4 (important), or 5 (very important) suggest that experts have reached a consensus regarding importance. The content validity indices of each item (I-CVI) and the overall scale (S-CVI) were calculated, and an S-CVI of more than 0.90 and an I-CVI of more than 0.78 were considered valid [30]. The validity of each item was determined through item analysis. We considered unfavorable floor or ceiling effects to be present if more than 15% of the individuals reached the highest or lowest score.

EFA was conducted using principal component analysis (PCA) as the extraction method and the varimax method as the rotation method to determine the factor structure of the questionnaire [31]. Items were deemed relevant if factor-loading coefficients exceeded 0.40 and extracted factors achieved an eigenvalue ≥ 1.0 [32]. A confirmatory factor analysis (CFA) was also performed to verify the results. The expected values of the indices recommended were as follows [33]: (a) chi-square divided by the degrees of freedom ≤ 3; (b) the root mean squared error of approximation (RMSEA) < 0.08; and (c) the comparative fit index (CFI), normed fit index (NFI), goodness-of-fit index (GFI) and Incremental Fit Index (IFI) > 0.90. It should be noted that the above model fit threshold values are simple guidelines and should not be interpreted as strict rules [21]. In addition, we calculated the composite reliability (CR) and average variance extracted (AVE) values for the factors to assess their convergent validity [34].

The internal consistency was calculated with the Cronbach’s alpha coefficient. A Cronbach’s alpha value of 0.7 or greater was considered satisfactory [35]. The split-half coefficient reliability was assessed by using half of the odd and even items. Test-retest reliability was assessed by using the intraclass correlation coefficient (ICC) [36]. ICC values of 0.60 to 0.80 were considered to indicate good reliability, and ICC values above 0.80 were considered to indicate excellent reliability [37].

## Results

### Refinement of NDASS

The expert review resulted in the rewording of 3 items and the deletion of 1 item, which was considered to be of low importance, leaving a total of 13 items. The S-CVI of the scale reached 0.975, which indicated excellent content validity. The I-CVIs were above 0.78 except for item 12 (“I can tolerate different perspectives on the internet”) (see Table 2). This item was deleted because of its low validity. In addition, the experts suggested modifying items with complex expressions and repetitive content. For example, the meaning of “reproduce” in item 1 belonged to item 2. Therefore, we removed the word “reproduce” in item 1. According to the experts, the description of item 4 was somewhat cumbersome, and we modified it to “I can use digital nursing equipment proficiently”. Similarly, the term “objectively” in item 5 was removed. The adjusted version of the NDASS was used for subsequent pilot study.

**Table 2:** The content validity of each item of NDASS

Item	I-CVI	Item	I-CVI	Item	I-CVI
1	1.000	6	1.000	11	1.000
2	1.000	7	0.929	^*#*^12	0.786
3	0.929	8	1.000	13	1.000
4	1.000	9	1.000	14	1.000
5	1.000	10	1.000		

*Note* ^*#*^indicates the entry needs to be deleted

The 30 nurses who participated in the pretesting were mainly from the ICU (64.6%), internal medicine (18.5%), surgery (9.2%), and other departments (7.7%). Some participants stated that the explanation of “digital skill” in the instructions was not simple enough, and the meaning of “digital” could not be understood clearly by reading the definition. After further explanation of the “digital”, the participants expressed their understanding and approval of the scale and items. We recorded participants’ suggestions during the pilot study and made modifications after discussion.

### Sample characteristics

The demographic data of the individuals included in the validation study are presented in Table 3. A total of 424 nurses who worked in the departments of internal medical (29.2%), surgery (14.6%), obstetrics and gynecology (4.0%), pediatrics (6.1%), emergency (6.4%), intensive care unit (14.4%), operating room (5.2%), rehabilitation unit (1.0%), or other (19.1%) units were included. The mean age was 32.11 years (SD = 5.48), and the mean years of service was 9.61 (SD = 6.05). Participants included 403 (95.0%) females and 21 (5.0%) males, the vast majority of whom obtained a bachelor’s degree or above (93.9%). Participants spent an average of 6.34 hours per day (SD = 4.30) using digital services, while only 27.1% had experience in digital courses or training. The average score of the NDASS (12 items) was 44.77 (SD = 9.49). There were significant differences in hospital level (*t* = 8.073, *p* < 0.001), type of unit (*F* = 7.312, *p* < 0.001), professional title (*F* = 3.175, *p* < 0.05), and received digital courses or training (*t* = 2.924, *p* < 0.01).

**Table 3:** General and occupation-related characteristics of the participants (*N* = 424)

Characteristics	*n* (%)	t/F
**Gender**		-0.874
Male	21(5.0)	
Female	403(95.0)	
**Educational attainment**		-0.446
College	26(6.1)	
Bachelor’s degree or above	398(93.9)	
**Hospital level**		8.073***
Grade-A tertiary hospital	350(82.5)	
Other	74(17.5)	
**Type of unit**		7.312***
Medicine unit	124(29.2)	
Surgical unit	62(14.6)	
Obstetrics/gynecology	17(4.0)	
Pediatrics	26(6.1)	
Emergency room	27(6.4)	
Intensive care unit	61(14.4)	
Operating room	22(5.2)	
Rehabilitation unit	4(1.0)	
Other	81(19.1)	
**Employment type**		-1.735
Permanent	62(14.6)	
Temporary	362(85.4)	
**Professional title**		3.175*
Nurse	57(13.4)	
Nurse practitioner	202(47.6)	
Nurse-in‐charge and above	165(39.0)	
**Received digital courses or training**		2.924**
Yes	115(27.1)	
No	309(72.9)	

*Note* *** *p* < 0.001, ** *p* < 0.01, * *p* < 0.05

### Item analysis

The values of the skewness and kurtosis of each item were examined, which ranged from − 0.84 to -0.14 and from − 0.35 to 1.58, respectively. The top 27% of the highest-scoring participants comprised the high group, and the lower 27% of the lowest-scoring participants comprised the low group. The mean score of each item in the two groups was subsequently compared using an independent samples t test to test the difference between the two groups, and the critical ratio (CR) of the item was obtained. The results showed that there was a statistically significant difference in the scores for each item between the high group and the low group (*p* < 0.001), and the CR value for each item was greater than 3, indicating that every item had good discrimination without the floor or ceiling effect. No items were deleted at this stage.

### Exploratory factor analysis

**Fig. 2:**
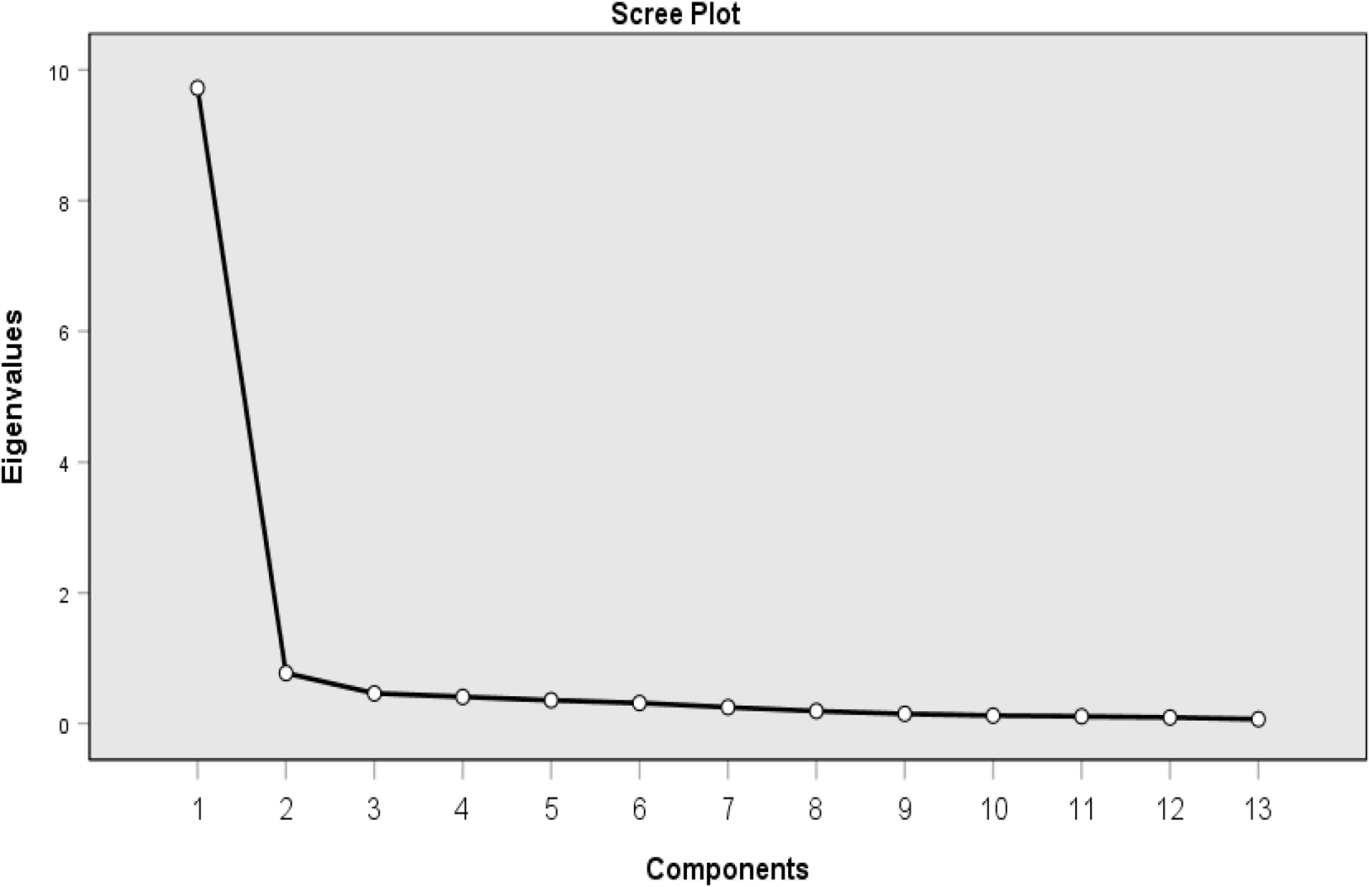
Scree plot of the NDASS (13 items)

The correlation matrix showed ample adequacy of the sample size (the Kaiser-Meyer-Olkin measure was 0.954, and the Bartlett test results (χ^2^ = 3156.793, *p* < 0.001) rejected the hypothesis of zero correlations. The scree plot (see Fig. 2) indicated that there was one factor. In addition, based on Kaiser’s criterion of extracting factors with eigenvalues greater than 1, a one-factor structure (Eigenvalue = 9.723) that explained 74.794% of the variance in the data were identified by the pattern matrix (see Table 4). Exploratory factor analysis of the 13 items produced factor loadings ranging from 0.786 to 0.918 (> 0.4).

**Table 4:** EFA-based factor loadings with varimax method of the NDASS (13 items)

Items	Factors 1
1	0.786***
2	0.835***
3	0.796***
4	0.817***
5	0.906***
6	0.865***
7	0.804***
8	0.898***
9	0.910***
10	0.887***
11	0.908***
12	0.918***
13	0.896***
**Eigenvalue**	9.723
**Cumulative variance (%)**	74.794%

*Note* *** *p* < 0.001

### Confirmatory factor analysis

**Fig. 3:**
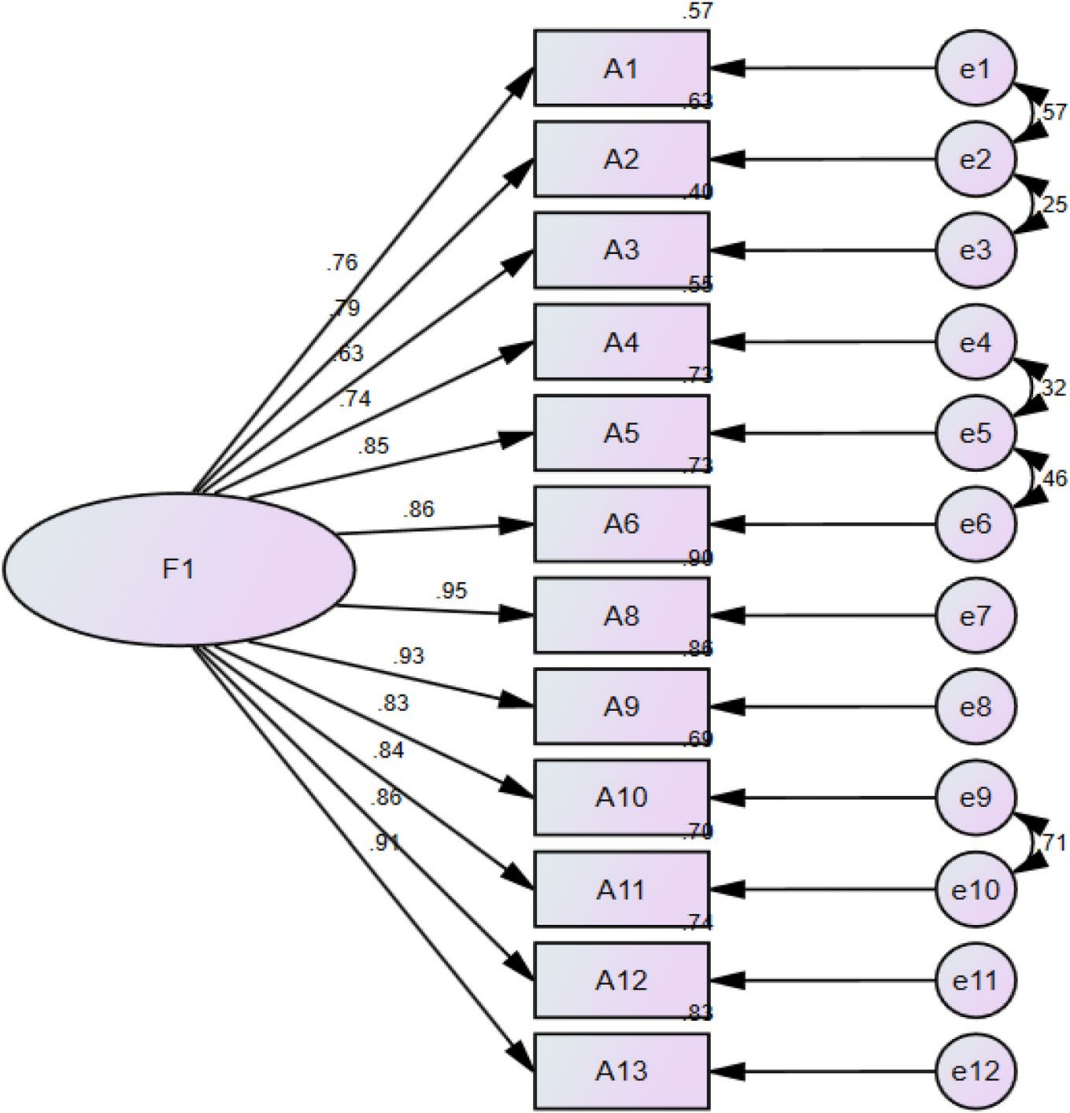
The schematic diagram of final standardized model fitting of the NDASS

A single-factor model was established according to the results of exploratory factor analysis (see Fig. 3). Model fit indices of each factor of the third version of the NDASS (13 items) were calculated. The CFA results revealed the following model fit indices: χ^2^/df = 7.602 (> 3), *p* < 0.001, RMSEA = 0.172 (> 0.08), CFI = 0.869 (< 0.9), NFI = 0.853 (< 0.9), and GFI = 0.741 (< 0.9). The results showed that the model and the data did not fit well (Table 5). During the model correction process, according to the modification index (M.I.) provided by AMOS 24.0, we found that item 7 had a greater residual correlation with item 8 (M.I. = 15.151) and had a lower factor loading than item 8. It is difficult to explain the negative correlation between item 7 and item 8 from a professional perspective. Additionally, upon careful examination of item 7 (I can express my thoughts clearly on the Internet), it became apparent that this item was more focused on measuring Internet skills. As a relatively basic skill, Internet skills reflect the limited level of digital application skills available to nurses. Thus, item 7 was removed from this model. In the modified model, the fit indices were excellent: the RMSEA was 0.076, less than 0.08; the GFI was 0.921; the CFI and IFI were both 0.979; and the NFI was 0.964, exceeding the benchmark of 0.90. Eventually, the single-factor model suitably fitted the survey data, and its application was testified to be appropriate for the population surveyed.

**Table 5:** The fitting indexes of confirmatory factor analysis of the NDASS (*N* = 224)

Index	Benchmark	Initial Model	Modified Model
χ^2^/df	< 3	7.602	2.295
RMSEA	< 0.08	0.172	0.076
GFI	> 0.9	0.741	0.921
CFI	> 0.9	0.869	0.979
NFI	> 0.9	0.853	0.964
IFI	> 0.9	0.870	0.979

To further confirm the validity of the fourth version of the NDASS (12 items), we checked the convergent validity. Convergent validity, also known as aggregate validity, might be tested by calculating the average variance extracted (AVE) and construct reliability (CR) values. The AVE value of this model was 0.694 (> 0.5), and the CR value was 0.964 (> 0.7). Both the AVE and CR values provided evidence of the convergent validity of the fourth version of the NDASS (12 items).

### Reliability

The Cronbach’s alpha of the final version of the NDASS was 0.968. The split-half reliability for the overall questionnaire was 0.935. The test-retest reliability according to the ICC was 0.740 (*p* < 0.001). The results indicated that the Nursing Digital Application Skill Scale has good reliability. The final version of the NDASS is shown in Table 6.

**Table 6:** Items of the final version of the NDASS (12 items)

Items
1. I can integrate existing digital content
2. I can create new digital content that meets expectations
3. I can protect intellectual property when creating digital content
4. I can use digital nursing equipment proficiently
5. I can use digital technology to analyze nursing problems
6. I can use statistical software to analyze nursing data
7. I can use digital technology to support nursing decision-making
8. I can use digital technology to promote nurse-patient relationships
9. I can use digital technology to collaborate with others
10. I can use digital technology to participate in social activities
11. I can use digital technology resources for continuous learning
12. I can apply digital technology to promote innovative nursing practices

## Discussion

With the integration of digital technologies in healthcare, scholars are considering the prerequisites for engagement with digital technologies and the importance of digital skills for effective engagement. In the current contribution, we proposed a new instrument for measuring nursing digital application skills through three steps: item generation, scale refinement, and scale validation. This five-point Likert scale with a single dimension is widely applicable. Its short length and concise presentation can help nurses make quick assessments. The results of the reliability and validity analysis showed that the 12-item NDASS was a reliable and valid instrument.

The tool was developed around the concept of nursing digital application skills and with reference to other digital skill assessment tools. For example, Fan [21] developed a digital skills questionnaire for Chinese college students, with a portion of the content being “use of digital means”, which is consistent with the theme of this study. However, because the respondents of the questionnaire were students, these items were related to learning. To this end, we established a research group composed of nursing education experts, clinical nurses, and doctoral and master’s students in nursing. The items were adapted based on the judgment of nursing knowledge to suit working professionals. A similar situation arose in the adaptation of the tools of Vanlaar [22]. Although the digital technology used in nursing was described, there was still a lack of nursing features in these items. An initial item pool for the NDASS was established by reviewing relevant literature and exploring the digital needs of the nursing profession. To refine the items, experts and clinical nurses were invited to evaluate the setting, description, and scoring rules of the items. Nursing authorities from universities and hospitals across different provinces in China ensured that the scale was refined under the guidance of professionalism and experience. The clinical nurses who participated in the pretest were also representatives from different hospitals and departments. The focus group interview confirmed that hospitals at different levels have varying degrees of digitalization, and clinical nurses’ involvement in digital technology also varies. Therefore, nurses from different hospitals were included to jointly complete the scale validation.

The success of an instrument largely depends on both the reliability and the validity of the measurement. The results of the expert review showed that the content validity index of most items was high, and the scale-level CVI reached 0.975, which met the recommended criteria [38]. The CVI of item 12 (“I can tolerate different perspectives on the internet”) was at a critical value. Based on expert opinions, the team believes that accommodating all perspectives is not a necessary criterion for digital skills. Instead, it is more crucial to utilize desirable recommendations, which are reflected in the other three items (“promote nurse-patient relationships”, “collaborate with others”, and “participate in social activities”). These three items are sufficient for describing nurses’ ability to communicate and socialize with different groups of people by using digital technologies. Therefore, item 12 was discarded.

Exploratory factor analysis indicated that only one principal component was extracted, accounting for 74.794% of the total variance. This result was consistent with that of a study that assessed the digital competence of health professionals in Swiss psychiatric hospitals [17]. This may also be related to the fact that the initial item pool was built without the module set up, and each item was rewritten based on concept. In contrast to this study, a study in Finland developed a five-factor model for assessing digital health competency. The model includes competence areas related to human-centered remote counseling, attitudes toward digital solutions as part of everyday work, skills in using information and communication technology, knowledge to utilize and evaluate different digital solutions, and ethical perspectives when using digital solutions [27]. The digital health model revolves around the connotation of competence, incorporating skills using remote counseling, information and communication technology, as well as attitudes and ethics. The NDASS aims to assess digital application skills and focuses on the different roles of the application of digital technologies in nursing work, which differs from the direction of the digital health competence scale. In addition, as a further contribution of this study, confirmatory factor analysis was performed to confirm the fitness of the single factor aligned with the general structure of the NDASS. The results of CFA indicated that the single-factor model with modification was considered a better fit, suggesting that the final version of the NDASS had good construct validity. Furthermore, the NDASS also had good convergent validity.

The nursing digital application scale displayed high internal consistency and split-half reliability as well as good test–retest reliability in our study, indicating that the scale was reliable and reproducible. Since cross-sectional data were used, computation of test-retest reproducibility might be an added advantage [39].

These results therefore suggest that the new scale is a suitable instrument for measuring skills in applying digital technologies among nurses, with acceptable reliability and validity. It applies to a broad group of nurses including clinical nurses, specialist nurses and nurse managers. NDASS focuses on the role of nurses in the clinic and describes their digital application skills in using digital technologies for work. Compared to previous general questions, the new scale provides respondents with the direction of thinking. Compared with scales that assess specific digital technologies, NDASS is more in line with the era of rapid digitalization. In addition, shorter and simpler questions can be more easily answered, further ensuring the reliability of the data. The Nursing Digital Application Skill Scale is a tool that can be used by clinical nurses to assess their digital skills daily. It can also guide nursing managers in developing training programs based on the differences in scores for various characteristics. For instance, the tool can be used to measure the digital gap between different populations (e.g., primary nurses and nurses-in-charge and above), enabling nursing managers to develop targeted interventions to address this gap. Furthermore, the tool can be used to assess the effectiveness of the training by measuring changes in scores before and after the training. In particular, the NDASS can be used to explore the relationships between digital skills and other variables in the subsequent studies.

Although the results of the validation of the NDASS are satisfactory, several limitations should be mentioned. For instance, nurses in our study were recruited via convenience sampling in Northwest China, which may have impacted the widespread generalization and application of NDASS to some degree. Nevertheless, the sample in our study covered the departments, years of service, and professional titles of nurses as much as possible, suggesting that the NDASS is understandable and acceptable to most nurses in China. Second, validity in online research is a well-studied concern [40]. We employed several strategies to improve the data validity, such as placing objectivity issues in the second half and conducting completion time checks, as we cannot control participants’ attention while answering questions. Third, criterion validity or predictive validity was not directly determined because a gold standard does not exist. Thus, the associations between digital application skills and other digital elements should be considered in future studies. Finally, while this single-factor short scale was considered to assess the most important digital skills required for clinical work after much discussion by the research group and the expert committee, other dimensions of the Digital Competence Framework for Citizens 2.2 (Information and data literacy, Safety, etc.) are also worthy of attention. Therefore, future research will focus on developing a multi-dimensional scale with a wider range of applicability.

## Conclusion

The digital skills of nurses are essential for the development of medical digitization. We proposed a succinct scale with 12 items to measure nurses’ digital skills in nursing work. The test results indicate that the scale is a reliable and effective instrument with excellent psychometric properties. The NDASS is replicable and applicable for the digital skill evaluation of nurses. In addition, nursing managers can use NDASS when designing nursing digital skill training.

## Data Availability

The datasets used and/or analysed during the current study are available from the corresponding author on reasonable request.

## Abbreviations

NDASS: Nursing Digital Application Skill Scale, ACE tool
SPSS: Statistical Package for the Social Sciences
AMOS: Analysis of Moment Structure
EFA: Exploratory Factor Analysis
CFA: Confirmatory Factor Analysis
SDs: Standard Deviations
CVI: Content validity index
I-CVI: Item level of content validity index
S-CVI: Scale level of content validity index
PCA: Principal component analysis
RMSEA: Root mean squared error of approximation
CFI: Comparative Fit Index
NFI: Normed Fit Index
GFI: Goodness-of-fit Index
IFI: Incremental Fit Index
CR: Critical Ratio
AVE: Average Variance Extracted
ICC: Intraclass correlation coefficient
